# Personal, health system, and geosocial disparities in appointment nonadherence at family medicine clinics in southcentral Pennsylvania, United States

**DOI:** 10.1002/jgf2.698

**Published:** 2024-05-06

**Authors:** Wen‐Jan Tuan, Ashley Weems, Shou Ling Leong

**Affiliations:** ^1^ Department of Family and Community Medicine, and Public Health Sciences, College of Medicine Pennsylvania State University Hershey Pennsylvania USA; ^2^ Department of Family and Community Medicine, College of Medicine Pennsylvania State University Hershey Pennsylvania USA

**Keywords:** continuity of care, health disparity, missed appointment

## Abstract

**Background:**

To assess the relationship between patients' demographic, health system‐related, and geosocial characteristics and the risk of missed appointments among patients in family medicine practice.

**Methods:**

The study was based on a retrospective cross‐sectional design using electronic health records and neighborhood‐level social determents of health metrics linked by geocoded patients' home address. The study population consisted of patients who had a primary care provider and at least one appointment at 14 family medicine clinics in rural and suburban areas in January–December 2022. Negative binomial regression was utilized to examine the impact of personal, health system, and geosocial effects on the risk of no‐shows and same‐day cancellations.

**Results:**

A total of 258,614 appointments were made from 75,182 patients during the study period, including 7.8% no‐show appointments from 20,256 patients. The analysis revealed that individuals in the ethnic minority groups were 1.24–1.65 times more likely to miss their appointments than their White counterpart. Females and English speakers had 14% lower risk for no‐show. A significant increase (32%–64%) in the odds of no‐shows was found among individuals on Medicaid and uninsured. Persons with prior history of no‐shows or same day cancellations were 6%–27% more likely to miss their appointments. The no‐show risk was also higher among people living in areas experiencing socioeconomic disadvantage.

**Conclusion:**

The risk of missed appointments is affected by personal, health system, and geosocial contexts. Future efforts aiming to reduce no‐shows could develop personalized interventions targeting the at‐risk populations identified in the analysis.

## Graphical abstract

Missed appointments (no‐shows and same day cancellations) are common problems in primary care settings that may lead to poor health outcomes, especially among individuals in the underserved populations. Our study identified that the risk of missed appointments is affected by personal, health system, and geosocial contexts. As we have better knowledge in barriers preventing patients from adhering to their appointments at primary care practices, future research is needed to develop personalized interventions to overcome these barriers and improve health equity.

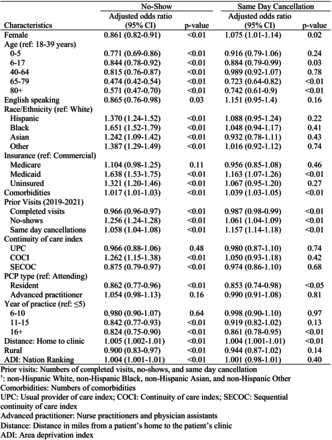

## 1 INTRODUCTION

Continuity of care has served as a cornerstone of primary care practice and the foundation of patient‐centered medical homes.^1^, ^2^ Better care continuum has been proven to lead to higher care quality, greater health outcomes, and less overall medical costs.^2^, ^3^ Yet, more than 15%–30% of patents missed their appointments,^4^ potentially costing the U.S. health systems over $150 billion dollars.^4^ There have also been concerns regarding an increased number of late cancellations which can disrupt the continuity of care for patients and interrupt clinical workflows for medical practices.^5^ The COVID‐19 pandemic has further exacerbated missed appointment rates, as the pandemic‐related anxiety and uncertainty increase.^6^ Despite great efforts in maintaining care continuum by building patient–provider relationships and offering more access options, many health systems continue to struggle with patients missing their appointments.

Missed appointments can be categorized into two types: no‐shows and same day cancellations. No‐shows refer to encounters when patients fail to attend scheduled appointments without prior notification or rescheduling.^7^ Same day cancellations are considered patients who cancel visits less than 24 h before their scheduled appointments. Health services research has often treated missed appointments by bundling no‐shows and same day cancellations together or focusing on no‐shows without same day cancellations.^8^, ^9^, ^10^ Yet, there is evidence suggesting that distinguishing no‐shows from same day cancellations can provide useful information for service operations and improving clinic utilization capacity, as the underlying causes of no‐shows and same day cancellations may vary.^9^ The prior literature found that missed appointments can be affected by patient characteristics, health system contexts, and socioeconomic factors.^7^ For instance, individuals who miss appointments tend to be young adults (aged 17–40 years),^11^ have multiple chronic conditions,^12^ experience socioeconomic challenges,^13^, ^14^ and live in deprived areas.^14^, ^15^ Moreover, race and ethnicity serve as predictors of missed appointments, as well as those with less comprehensive insurance coverage were more likely to miss appointments.^16^

Missed appointments are a serious problem in primary care which provides essential services to underserved chronically ill populations, increasing disparities among these populations already experiencing health inequities.^17^, ^18^ The objective of the study was to identify personal, health system‐related, and neighborhood‐level social determinant of health (SDOH) factors influencing the occurrence of missed appointments among patients managed by primary care clinicians at Penn State Health's family medicine clinics. The results of the analysis would provide additional insights into the design and implementation of the future population‐based intervention to reduce missed appointments among patients managed at primary care settings.

## 2 METHODS

### 2.1 Study design and population

This study utilized a retrospective cross‐sectional design using electronic health records (EHRs) from the enterprise data warehouse of a large health system and academic medical center in southcentral Pennsylvania, United States. To be included in the analysis, patients must have a primary care provider (PCP) between January 2022 and December 2022 at one of the 14 family medicine clinics of the health system. To ensure that patients were actively managed by a PCP in the context of ongoing care, individuals eligible in the study must have at least one appointment or visit event at family medicine clinics anytime in January–December 2022. Patients deceased or living outside of Pennsylvania were excluded from the study. This study was approved by the academic medical center's Institutional Review Board.

### 2.2 Outcome measures

The primary outcomes of interest were the no‐show count and the same day cancellation count of the patient. The no‐show count was computed as the total number of no‐show events for each patient in 2022. The same day cancellation count was calculated as the total number of cancellations within 24 h prior to the scheduled appointment in the analysis period. Because the majority of patients completed their appointments, most people were expected to have a value of zero for the no‐show measure and the same day cancellation measure.

### 2.3 Patient characteristics

The baseline characteristics of the analysis consisted of three domains: patient characteristics, healthcare utilization factors, and geosocial contexts. Patient characteristics included gender, age, race, ethnicity, insurance type, and comorbid conditions. Patients' age was assigned to six groups: 0–5, 6–17, 18–39, 40–64, 65–79, and 80+ years. The ethnicity of the study population was classified into two ethnic groups: Hispanic and non‐Hispanic. Patients' race was categorized into four groups: White, Black, Asian, and Other race categories. Persons reported as American Indian, Pacific Islanders, and unknown races were aggregated into the Other race category due to their small numbers. The analysis categorized patient's insurance type to one of the four payer categories: commercial, Medicare, Medicaid, and other (uninsured and self‐pay). Patient's health complexity was assessed by applying the Elixhauser comorbidity method using a set of 29 medical, psychiatric, and lifestyle‐related health conditions.^19^, ^20^ The study used the number of diseases in the Elixhauser measure to represent patient complexity because the simple count measurement of unique medical conditions is a reliable predictor of healthcare utilization similar to other complex comorbidity indices.^21^

### 2.4 Healthcare utilization characteristics

The analysis also incorporated healthcare utilization‐related characteristics, such as the history of prior completed and missed appointments, continuity of care indices, the type of PCP (attending physician, resident physician, physician assistant/nurse practitioner), and PCP's years of practice. The history of prior appointments was measured by three variables: the numbers of completed visits, no‐shows, and same day cancellations in the past 3 years. The analysis applied the 3‐year look‐back criteria because this timeframe has been commonly used to determine the level of care continuum in primary care research.^22^, ^23^ Moreover, three common continuity indices were computed to assess the degree of continuity, specific to the density, dispersion, and sequential nature of care continuity in the past 3 years.^24^ The usual provider of care index (UPC) is calculated as the proportion of visits to the patient's PCP over among visits at the PCP's clinic. The index represents the “density” of the PCP–patient interaction, with a value ranging from 0 (no visits to PCP) to 1 (all visits made to PCP). The continuity of care index (COCI) is the weighted percentage of visits to distinct providers. It measures the “dispersion” of visits among all caretakers seen by a patient. The index ranges from 0 (each visit made to a different provider) to 1 (all visits made to the same provider). The sequential continuity of care index (SECOC) computes the proportion of visits made to the same caretakers between two adjacent visits. The index illustrates the sequential nature of visits, with a value ranges from 0 (no same providers at the adjacent visits) to 1 (all visits made to the same provider). Each index represents a unique aspect of continuity and provides complementary information.

### 2.5 Geosocial contexts

Moreover, patients' home address was converted to geographic coordinates using ArcGIS Pro Version 2.8 (ESRI Inc, Redlands, CA) and then matched to a specific census block group code. The distance from the patient's home to their primary care clinic was estimated in miles using the Euclidean method to account for any sensitivity to distance barrier. The rural status of the patient was determined by linking geographic coordinates to the census urban and rural classification mapping file.^25^ The score of the area deprivation index (ADI) was obtained at the census block group level to serve as the proxy of the socioeconomic status of the patient in the neighborhood context.^26^ ADI is a composite measure of 17 census‐based social determinants which factors in domains of poverty, education, employment, and housing quality.^26^

### 2.6 Statistical analysis

Descriptive statistics were computed to describe sample characteristics by outcome measure. Differences in continuous variables were compared using the *t*‐test. Proportion differences in categorical variables were evaluated using the chi‐square test. Negative binomial regression was applied to assess the impact of the baseline characteristics on the likelihoods of no‐shows and same day cancellations, respectively. Because the distributions of the no‐show and same day cancellation events were expected to be sparse and positively skewed with a long right tail, negative binomial regression with a log link transformation would be a good modeling approach to address the over‐dispersed nature of count measures with the conditional variance exceeding the conditional mean.^27^, ^28^ The regression analysis was computed using the maximum likelihood estimation method, which provided the adjusted odds ratio (aOR) and 95% CI of each variable. The significance level was determined based on two‐tailed *p*‐value <0.05. All statistical analyses were performed using PROC GENMOD procedure (Version 9.4 SAS Institute Inc., Carey, NC).

### 2.7 Sensitivity analysis

Younger and older patients may need assistance to attend their appointments, due to access, mobility, or cognitive limitations. Thus, their likelihood to adhere appointments could depend on the characteristics of their caretakers. To determine the robustness of the study's assessment, we performed a sensitivity analysis on the adult population whose age was in the range of 18–79 years. The analysis examined the extent to which results were affected by changes in the study populations, providing additional insight into the validity of the study funding.

## 3 RESULTS

### 3.1 Overall population summary

**FIGURE 1:**
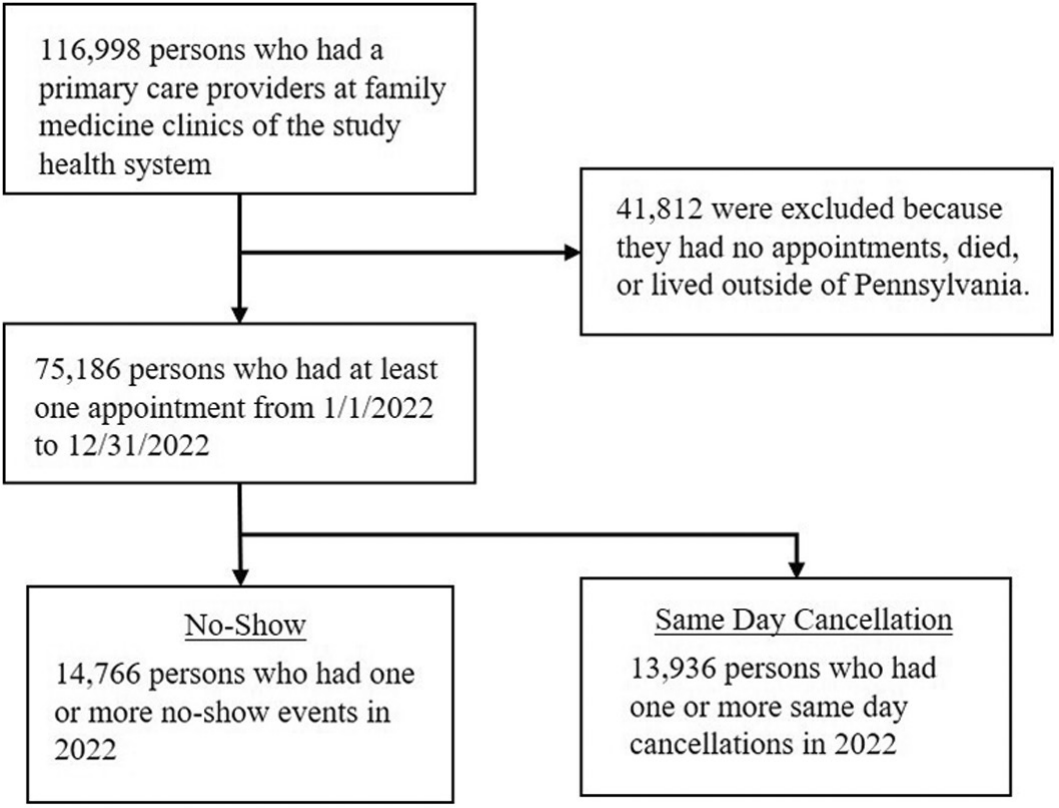
Flow diagram of the population inclusion criteria. To be included in the study population, patients (1) had a primary care provider at a family medicine clinic, (2) had at least one appointment in January–December 2022, and (3) lived in Pennsylvania.

**FIGURE 2:**
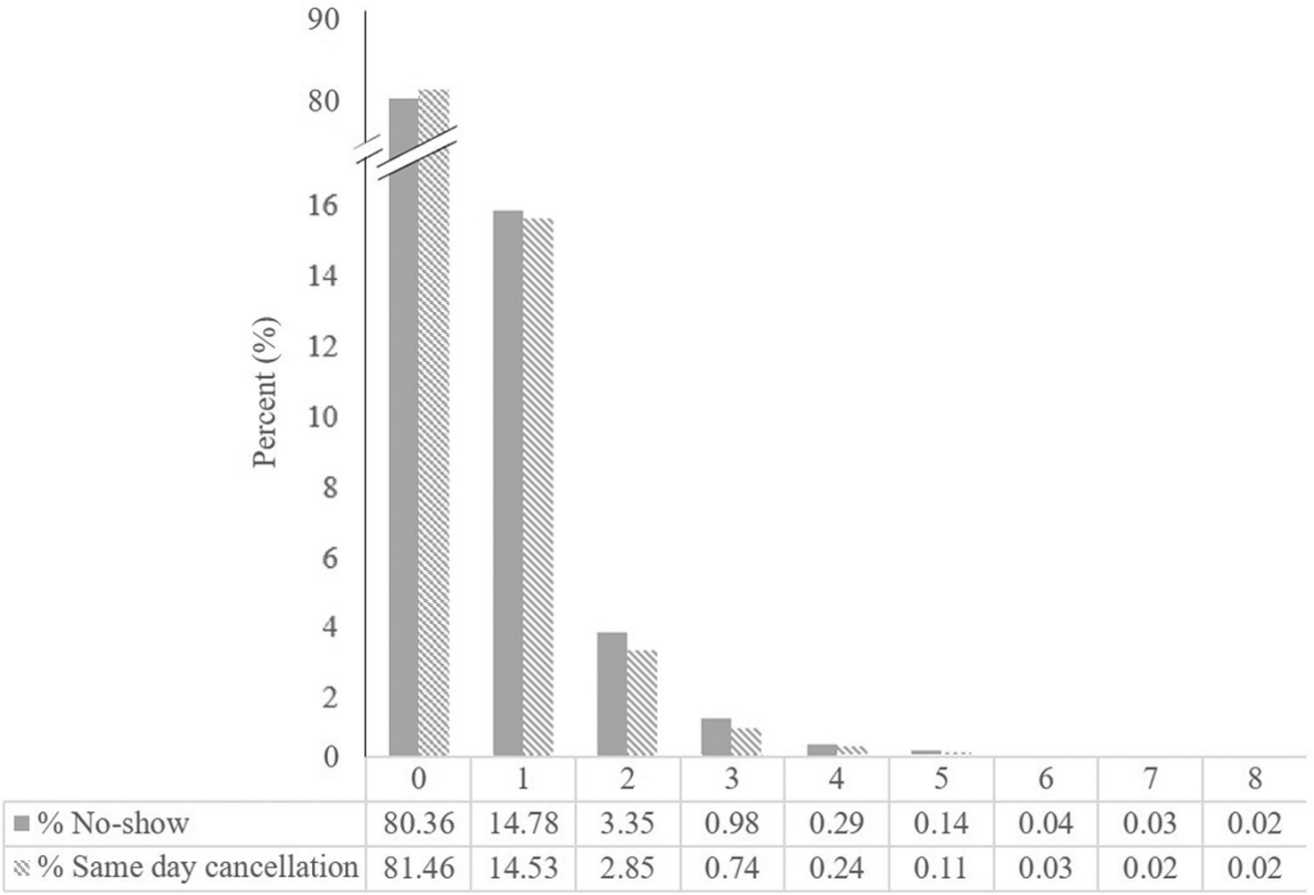
Distribution of the percentage of patients by the numbers of no‐shows and same day cancellations.

The analysis consisted of 75,186 patients with 258,590 appointments scheduled in January–December 2022, including 85.1% appointments completed, 7.8% with a no‐show status, and 7.1% appointments canceled within 24 h. Figure 1 provides the flow diagram of the study sample selection. Of 75,186 patients, about 20% (14,776) had no‐show events and 19% (13,936) had same day cancellations. Figure 2 shows that the majority of patients had only one missed appointment events, and the percentage of patients declined as the number of missed appointments increased, suggesting a negative binomial distribution. The characteristics of the study populations with no‐shows or same day cancellations are summarized in Table 1. Overall, a greater percentage of patients in the no‐show and same day cancellation group was younger, non‐English speaking, ethnic minorities, publicly insured, and sicker. The percentage of persons with no‐shows or same day cancellations also varied by the level of continuity of care, provider type, provider year of practice, and home location.

**TABLE 1:** Population characteristics by no‐show and same day cancellation.

Characteristics	No‐show			Same day cancellation		
	Yes, *N* = 14,766	No, *N* = 60,420	*p*‐Value	Yes, *N* = 13,936	No, *N* = 61,250	*p*‐Value
Female, *n* (%)						
Yes	8364 (56.6)	34,207 (56.6)	0.95	8627 (61.9)	33,944 (55.4)	<0.01
No	6402 (43.4)	26,213 (43.4)		5309 (38.1)	27,306 (44.6)	
Age, *n* (%)						
0–5	1106 (7.5)	2741 (4.5)	<0.01	865 (6.2)	2982 (4.9)	<0.01
6–17	1708 (11.6)	6397 (10.6)		1232 (8.8)	6873 (11.2)	
18–39	5092 (34.5)	14,502 (24)		3928 (28.2)	15,666 (25.6)	
40–64	4865 (32.9)	20,729 (34.3)		5037 (36.1)	20,557 (33.6)	
65–79	1352 (9.2)	11,354 (18.8)		1945 (14)	10,761 (17.6)	
80+	643 (4.4)	4697 (7.8)		929 (6.7)	4411 (7.2)	
English speaking, *n* (%)						
Yes	13,979 (94.7)	59,063 (97.8)	<0.05	13,491 (96.8)	59,551 (97.2)	<0.01
No	787 (5.3)	1357 (2.2)		445 (3.2)	1699 (2.8)	
Hispanic, *n* (%)						
Yes	1163 (7.9)	2331 (3.9)	<0.01	792 (5.7)	2702 (4.4)	<0.01
No	13,603 (92.1)	58,089 (96.1)		13,144 (94.3)	58,548 (95.6)	
Race/Ethnicity^a^, *n* (%)						
Hispanic	1163 (7.9)	2331 (3.9)	<0.01	792 (5.7)	2702 (4.4)	<0.01
White	8313 (56.3)	45,670 (75.6)		9611 (69.0)	44,372 (72.4)	
Black	2261 (15.3)	3461 (5.7)		1347 (9.7)	4375 (7.1)	
Asian	739 (5.0)	2173 (3.6)		510 (3.7)	2402 (3.9)	
Other	2290 (15.5)	6785 (11.2)		1676 (12)	7399 (12.1)	
Health insurance, *n* (%)						
Commercial	6192 (41.9)	34,627 (57.3)	<0.01	6719 (48.2)	34,100 (55.7)	<0.01
Medicare	1698 (11.5)	11,365 (18.8)		2315 (16.6)	10,748 (17.5)	
Medicaid	5802 (39.3)	9693 (16)		3905 (28)	11,590 (18.9)	
Uninsured	1074 (7.3)	4735 (7.8)		997 (7.2)	4812 (7.9)	
Comorbidities, *μ* (*σ*)	3 ± 3.1	2.6 ± 2.8	<0.01	3.4 ± 3.4	2.5 ± 2.7	<0.01
Prior visits (2019–2021), *μ* (*σ*)						
Completed visits	7.3 ± 7.1	7.0 ± 6.6	<0.01	8.6 ± 7.9	6.7 ± 6.3	<0.01
No‐Show visits	1.1 ± 1.6	0.3 ± 0.7	<0.01	0.7 ± 1.4	0.4 ± 0.9	<0.01
Canceled in 24 h	0.9 ± 1.6	0.4 ± 0.9	<0.01	1.0 ± 1.7	0.4 ± 0.8	<0.01
Continuity of care index, *μ* (*σ*)						
UPC	0.3 ± 0.3	0.4 ± 0.3	<0.01	0.3 ± 0.3	0.4 ± 0.3	<0.01
COCI	0.4 ± 0.4	0.5 ± 0.4	<0.01	0.4 ± 0.4	0.5 ± 0.4	<0.01
SECOC	0.6 ± 0.3	0.6 ± 0.3	<0.01	0.6 ± 0.3	0.6 ± 0.4	0.15
PCP type, *n* (%)						
Physician	7595 (51.4)	35,987 (59.6)	<0.01	7852 (56.3)	35,730 (58.3)	<0.01
Resident	1768 (12)	5178 (8.6)		1316 (9.4)	5630 (9.2)	
Advanced practitioner	5403 (36.6)	19,255 (371.9)		4768 (34.2)	19,890 (32.5)	
Years of practice, *n* (%)						
0–5	5450 (36.9)	17,569 (29.1)	<0.01	4462 (32)	18,557 (30.3)	<0.01
6–10	2636 (17.9)	9603 (15.9)		2494 (17.9)	9745 (15.9)	
11–15	2355 (15.9)	9814 (16.2)		2285 (16.4)	9884 (16.1)	
16+	4325 (29.3)	23,434 (38.8)		4695 (33.7)	23,064 (37.7)	
Distance: Home to clinic, *μ* (*σ*)	7.8 ± 10.9	7.3 ± 10.6	<0.01	7.8 ± 10.6	7.3 ± 10.7	<0.01
Rural area, *n* (%)						
Yes	2254 (15.3)	12,780 (21.2)	<0.01	2499 (17.9)	12,535 (20.5)	<0.01
No	12,512 (84.7)	47,640 (78.8)		11,437 (82.1)	48,715 (79.5)	
ADI: Nation Ranking, *μ* (*σ*)	55.5 (18.8)	49.8 (16.8)	<0.01	52.9 (17.8)	50.4 (17.3)	<0.01

*Note*: *n*: count; %: percent; μ: mean; and σ: standard deviation. Prior visits: numbers of completed visits, no‐shows, and same day cancellation. Advanced practitioner: Nurse practitioners and physician assistants. Distance: Distance in miles from a patient's home to the patient's clinic.

Abbreviations: ADI: Area deprivation index; COCI: Continuity of care index; PCP: Primary care provider; SECOC: Sequential continuity of care index; UPC: Usual provider of care index.

^a^ Non‐Hispanic White, non‐Hispanic Black, non‐Hispanic Asian, and non‐Hispanic Other.

### 3.2 No‐show

Table 2 provides the adjusted odds ratio of no‐show risk from negative binomial regression, controlled for personal, healthcare‐related, and geosocial factors. Females were 14% less likely to miss their appointments than their male counterpart (aOR = 0.86, 95% CI 0.82–0.91). Most of the age groups were 16%–53% less likely to have no‐show events, compared to persons aged 18–39 years. There was a 13% decrease in the odds of no‐shows among English speakers (aOR = 0.87, 95% CI 0.76–0.98). Ethnic minority groups were found to be 1.24–1.65 more likely to miss their appointments than their White counterpart. Compared to those commercially insured, a significant increase in the odds of no‐shows was found among individuals on Medicaid (aOR = 1.64, 95% CI 1.53–1.75) and uninsured (aOR = 1.32, 95% CI 1.20–1.46), but no difference was found in Medicare patients.

**TABLE 2:** Results of the negative binomial regression analysis.

Characteristics	No‐show		Same day cancellation	
	Adjusted odds ratio (95% CI)	*p*‐Value	Adjusted odds ratio (95% CI)	*p*‐Value
Female	0.861 (0.82–0.91)	<0.01	1.075 (1.01–1.14)	0.02
Age (ref: 18–39 years)				
0–5	0.771 (0.69–0.86)	<0.01	0.916 (0.79–1.06)	0.24
6–17	0.844 (0.78–0.92)	<0.01	0.884 (0.79–0.99)	0.03
40–64	0.815 (0.76–0.87)	<0.01	0.989 (0.92–1.07)	0.78
65–79	0.474 (0.42–0.54)	<0.01	0.723 (0.64–0.82)	<0.01
80+	0.571 (0.47–0.70)	<0.01	0.742 (0.61–0.9)	<0.01
English speaking	0.865 (0.76–0.98)	0.03	1.151 (0.95–1.4)	0.16
Race/Ethnicity (ref: White^a^)				
Hispanic	1.370 (1.24–1.52)	<0.01	1.088 (0.95–1.24)	0.22
Black^a^	1.651 (1.52–1.79)	<0.01	1.048 (0.94–1.17)	0.41
Asian^a^	1.242 (1.09–1.42)	<0.01	0.932 (0.78–1.11)	0.43
Other^a^	1.387 (1.29–1.49)	<0.01	1.016 (0.92–1.12)	0.74
Insurance (ref: Commercial)				
Medicare	1.104 (0.98–1.25)	0.11	0.956 (0.85–1.08)	0.46
Medicaid	1.638 (1.53–1.75)	<0.01	1.163 (1.07–1.26)	<0.01
Uninsured	1.321 (1.20–1.46)	<0.01	1.067 (0.95–1.20)	0.27
Comorbidities	1.017 (1.01–1.03)	<0.01	1.039 (1.03–1.05)	<0.01
Prior visits (2019–2021)				
Completed visits	0.966 (0.96–0.97)	<0.01	0.987 (0.98–0.99)	<0.01
No‐shows	1.256 (1.24–1.28)	<0.01	1.061 (1.04–1.09)	<0.01
Same day cancellations	1.058 (1.04–1.08)	<0.01	1.157 (1.14–1.18)	<0.01
Continuity of care index				
UPC	0.966 (0.88–1.06)	0.48	0.980 (0.87–1.10)	0.74
COCI	1.262 (1.15–1.38)	<0.01	1.050 (0.93–1.18)	0.42
SECOC	0.875 (0.79–0.97)	<0.01	0.974 (0.86–1.10)	0.68
PCP type (ref: Attending)				
Resident	0.862 (0.77–0.96)	<0.01	0.853 (0.74–0.98)	<0.05
Advanced practitioner	1.054 (0.98–1.13)	0.16	0.990 (0.91–1.08)	0.81
Year of practice (ref: ≤5)				
6–10	0.980 (0.90–1.07)	0.64	0.998 (0.90–1.10)	0.97
11–15	0.842 (0.77–0.93)	<0.01	0.919 (0.82–1.02)	0.13
16+	0.824 (0.75–0.90)	<0.01	0.861 (0.78–0.95)	<0.01
Distance: Home to clinic	1.005 (1.002–1.01)	<0.01	1.004 (1.001–1.01)	<0.01
Rural	0.900 (0.83–0.97)	<0.01	0.944 (0.87–1.02)	0.14
ADI: Nation Ranking	1.004 (1.001–1.01)	<0.01	1.001 (0.98–1.01)	0.40

*Note*: Prior visits: Numbers of completed visits, no‐shows, and same day cancellation. Comorbidities: Numbers of comorbidities. Advanced practitioner: Nurse practitioners and physician assistants. Distance: Distance in miles from a patient's home to the patient's clinic.

Abbreviations: ADI: Area deprivation index; COCI: Continuity of care index; SECOC: Sequential continuity of care index; UPC: Usual provider of care index.

^a^ Non‐Hispanic White, non‐Hispanic Black, non‐Hispanic Asian, and non‐Hispanic Other.

Patients who had resident physicians as their PCP were 14% less likely to miss their appointments (aOR = 0.86, 95% CI 0.77–0.96). PCP who had 10 or more years of practice was found to have 16%–18% lower no‐show risk, compared to PCP practicing less than 5 years. Patients with more completed visits had lower odds of no‐shows (aOR = 0.97, 95% CI 0.96–0.97), while persons with prior history of missed appointments were 6%–27% more likely to miss their appointments. A lower no‐show risk was also found among patients with greater index values of SECOC (aOR = 0.88, 95% CI 0.79–0.97), but a higher risk of no‐shows was found among patients with the greater level of COCI (aOR = 1.26, 95% CI 1.15–1.38).

Moreover, the risk of no‐shows increased by approximately 0.5% as the patient's home–clinic distance increased every one mile (aOR = 1.005, 95% CI 1.002–1.01). The analysis showed that the odds of no‐shows also increased 0.4% as the ADI score increased one unit (aOR = 1.004, 95% CI 1.001–1.01), suggesting associations between no‐shows, poverty, and social disadvantage. Interestingly, individuals living in rural communities were 10% less likely to miss their appointments (aOR = 0.90, 95% CI 0.83–0.97).

### 3.3 Same day cancellation

Unlike no‐shows, females were about 8% more likely to cancel their appointments within 24 h before their scheduled visits than their male counterpart (aOR = 1.08, 95% CI 1.01–1.14). Compared to those aged 18–39 years, individuals in the 6–17, 65–79, and 80+ groups showed 12%–28% lower odds of same day cancellations, but differences were not found in the rest of the age groups. Only Medicaid patients had higher odds of cancellations than individuals with commercial insurance (aOR = 1.16, 95% CI 1.07–1.26). Similar to no‐shows, individuals with more completed visits had lower odds of same day cancellations (aOR = 0.99, 95% CI 0.98–0.99), and persons with prior history of no‐shows or same day cancellations were 6%–16% more likely to cancel their appointments within 24 h prior to their scheduled appointments. Patients whose PCP was a resident physician were 15% less likely to cancel their appointments (aOR = 0.85, 95% CI 0.74–0.98). A lower no‐show probability was only found in PCP who had 16 or more years of practice (aOR = 0.86, 95% CI 0.78–0.95). The risk of same day cancellations increased by 0.4% as the patient's home–clinic distance increased one mile (aOR = 1.004, 95% CI 1.002–1.007). Different from the no‐show, the likelihood of same day cancellations was not associated with whether patients resided in rural areas or with the ADI of the neighborhood.

### 3.4 Sensitivity analysis

The sensitivity analysis shows that the effects of the regression model for the adult population (ages 18–79) were consistent with those of the main analysis (see Table S1 in supplemental materials). It assures that the statistical modeling outcomes did not vary among different populations.

## 4 DISCUSSION

This research provides an innovative approach to assess the risks of no‐shows and same day cancellations by integrating patient‐level EHR data with the geosocial contexts, offering novel insights on the impact of the socioeconomic conditions on missing appointments in primary care settings. Our analysis found approximately 20% of the patient population accounted for all the missed or canceled appointments. The patient's prior appointment completion status was found to be strongly associated with no‐shows and same day cancellations in this study. Individuals with a history of more missed appointments tend to miss or cancel their future appointments. Our analysis shows that, of patients with no‐shows or same day cancellations, over 25% of them repeatedly missed their appointments. The current practice is to remove these patients from a provider's patient panel after a certain number of missed appointments; however, termination of care could affect patients' access to essential care and should be considered the last resort. To minimize the disruption to the clinical workflow, some practices double‐book these patients with a short visit or give them an appointment at the end of the day where the missed appointment would lessen the disruption.^29^, ^30^

The study identified a number of characteristics associated with the likelihood of no‐shows and same day cancellations. Consistent with prior studies, we found that women were less likely to miss their appointments than men. This may be explained by women having greater needs for regular physical examinations, as well as more awareness and interest in their health. Lower no‐show rates for elderly patients and for those with chronic illness could reflect a greater sense of physical vulnerability resulting in stronger inclinations to pay attention to their own health and to seek health‐related information. Similarly, children were less likely to miss their appointment due to additional care needs in both infant and child development stages. Patients in the age groups 18–39 years are at the highest risk of missing their appointments, possibly due to work or school conflict.

Our analysis showed greater risk of no‐shows in minority patients.^31^ This could be contributed to a variety of reasons, such as poor experience in interacting with healthcare systems, disrespect for their beliefs, and misunderstanding of appointment processes.^32^ The study also identified health insurance as an important predictor for missing appointments. Individuals who were uninsured or underinsured seemed more likely to miss their appointments. The finding is consistent with the prior literature,^7^, ^33^ suggesting that nonclinical support should be considered to help those patients obtain necessary health insurance coverage for better appointment adherence.

Contrary to general beliefs, patients who experienced a greater continuity of care in our study did not always have lower missed appointment rates. Overall, the care continuum did not affect same day cancellation rates. Our data did not find associations between no‐shows and whether a visit was rendered by the patient's PCP, as demonstrated through the UPC index. The greater dispersion of visits showed higher no‐show rates, as demonstrated through the COCI. However, after accounting for the sequence of visits in which providers were seen, the greater continuum was found to have the lower no‐show rate, as per the SECOC index. In general, the results suggest that patients be less likely to miss their appointments when they are seen by the same providers over time.

The study also found lower odds of missed appointments among patients with a PCP in their middle or advanced career phase. This is possibly because patients tend to have better care experience, satisfaction, and a trusting relationship with PCPs who had more years of practice, motivating patients to adhere to their appointments.^34^ Interestingly, our analysis found that patients whose PCP was a resident physician were less likely to miss their appointments overall.

Missed appointments are also associated with structural barriers, such as travel distance to clinic and transportation availability.^16^, ^35^, ^36^ Greater risks of no‐shows and same day cancellations were also found among patients living farer away from their primary care clinic in our study. However, the odds ratio of the distance factor was close to one. The small effect size might not provide enough clinical or practical relevance for health systems to develop actionable interventions. Prior literatures also suggested that individuals residing in rural areas experience a higher no‐show rates.^7^ Yet, our analysis found that patients living in rural areas had a lower odds of no‐shows. This could be in part because the health system has a large network of clinics located in rural communities, which improve access for people living in rural areas.

Our finding also revealed that patients living in neighborhoods experiencing greater economic hardship had increased risk of missing their appointments, despite small effect sizes. Poverty has been considered a key barrier for access to healthcare and adherence to clinic appointments.^13^, ^37^ Individuals or families experiencing financial difficulty could miss or cancel their doctor appointments because of work conflict,^38^ lack of transportation,^36^, ^38^ and family obligation (child or elderly care). The literature has also shown that economic hardship disproportionately exists among individuals in the ethnic minority population, which could be in part contributed to high no‐show rates in this group as shown in the finding of the study. The increased risk that both minority and socioeconomically deprived patients are more likely to miss appointments is particularly concerning when these appointments involve preventive care or chronic disease management. This pattern of nonattendance can amplify health inequalities and thus deserves further attention.

### 4.1 Limitation

Our study included several limitations. First, despite a large sample size, the study was based on a single academic healthcare center, potentially limiting the results' generalizability. Second, the analysis did not include individual‐level geosocial indicators because the data were not collected in the EHRs. While the census statistics offers reliable and representative information for individuals living in the neighborhood, these data may not capture all the variations among individuals, possibly diluting predictive power.^31^ Third, because the patient was the unit of analysis of the study, predictive modeling in this analysis did not incorporate encounter‐level information, such as scheduled lead time, weather, and other macro‐level conditions which could also affect no‐shows.^7^, ^39^, ^40^ Future research is needed to evaluate the risk of no‐shows and same day cancellations at the encounter level. Fourth, although the timeframe of this study was after the peak of the COVID‐19 pandemic, the overall missed appointment rate might be higher partly due to patients' fear of COVID‐19 infection. Lastly, some geosocial factors were found to have small but statistically significant effects on missed appointments, potentially due to the large sample size. It simply means that the association was unlikely to have occurred by chance; however, the effect size might be too small to be conveyed into actionable plans in the real‐world healthcare practice. Extra caution should be taken to determine whether those effects were clinically meaningful to the patients. Additional research should be conducted in a future timeframe to validate the analysis outcomes.

## 5 CONCLUSION

No‐shows and same day cancellations do not only interrupt clinical workflows at primary care practices but also lead to delays in essential patient care, compromised health outcomes. Our study identified individuals with higher risk of missed appointments are likely to be male, minority, uninsured, in the young adulthood, sicker, or experiencing socioeconomic challenges. A substantial portion of patients with missed appointments had prior history of multiple no‐shows or same day cancellations. Our analysis suggests that no‐shows and same day cancellations could be two distinct phenomena contributed by different types of personal, system, and geographic factors. As we have better knowledge in barriers preventing patients from adhering to their appointments at primary care practices, future research is needed to develop personalized interventions to overcome these barriers and improve health equity.

## AUTHOR CONTRIBUTIONS

All authors were involved in developing and approved the manuscript. WJT conducted literature review, extracted EHR data, performed statistical analysis, and contributed to writing the manuscript. AW contributed to literature review, data analysis, and writing the manuscript. SLL involved in study design, data validation, interpretation, and writing the manuscript.

## FUNDING INFORMATION

None reported.

## CONFLICT OF INTEREST STATEMENT

The authors declare no conflict of interest.

## ETHICS STATEMENT

Ethics approval statement: The research was approved by Pennsylvania State University's institutional review board (ID: STUDY00022245).

Patient consent statement: Patient consent for publication is not required.

## Supporting information


Table S1.
