# Supplementary material for: Personal, health system, and geosocial disparities in appointment nonadherence at family medicine clinics in southcentral Pennsylvania, United States
Source: J Gen Fam Med. 2024 May 6;25(4):214–23. doi: 10.1002/jgf2.698 (PMC11221050; doi:10.1002/jgf2.698)
Supplement: Supplementary file 1 — Table S1. [file JGF2-25-214-s001.docx]

**Table S1. Results of the Sensitive Analysis**

|  | No-Show | |  | Same Day Cancellation | |
| --- | --- | --- | --- | --- | --- |
| Characteristics | Adjusted odds ratio (95% CI) | p-value |  | Adjusted odds ratio (95% CI) | p-value |
| Female | 0.825 (0.78-0.88) | <0.01 |  | 1.092 (1.02-1.17) | 0.01 |
| Age (ref: 18-39 years) |  |  |  |  |  |
| 40-64 | 0.808 (0.76-0.86) | <0.01 |  | 0.997 (0.92-1.07) | 0.93 |
| 65-79 | 0.471 (0.41-0.54) | <0.01 |  | 0.729 (0.64-0.83) | <0.01 |
| English speaking | 0.785 (0.68-0.91) | <0.01 |  | 1.172 (0.93-1.47) | 0.17 |
| Race/Ethnicity (ref: White) |  |  |  |  |  |
| Hispanic | 1.411 (1.25-1.59) | <0.01 |  | 1.094 (0.94-1.28) | 0.26 |
| Black | 1.638 (1.49-1.80) | <0.01 |  | 1.078 (0.95-1.22) | 0.25 |
| Asian | 1.244 (1.07-1.45) | <0.01 |  | 0.972 (0.80-1.18) | 0.77 |
| Other | 1.350 (1.24-1.47) | <0.01 |  | 1.018 (0.91-1.13) | 0.75 |
| Insurance (ref: Commercial) |  |  |  |  |  |
| Medicare | 1.079 (0.95-1.23) | 0.25 |  | 0.967 (0.85-1.1) | 0.61 |
| Medicaid | 1.637 (1.52-1.76) | <0.01 |  | 1.180 (1.08-1.29) | <0.01 |
| Uninsured | 1.294 (1.16-1.44) | <0.01 |  | 1.060 (0.94-1.20) | 0.36 |
| Comorbidities | 1.021 (1.01-1.03) | <0.01 |  | 1.039 (1.02-1.05) | <0.01 |
| Prior Visits (2019-2021) |  |  |  |  |  |
| Completed visits | 0.966 (0.96-0.97) | <0.01 |  | 0.986 (0.98-0.99) | <0.01 |
| No-shows | 1.250 (1.23-1.27) | <0.01 |  | 1.063 (1.03-1.09) | <0.01 |
| Same day cancellations | 1.056 (1.03-1.08) | <0.01 |  | 1.154 (1.13-1.17) | <0.01 |
| Continuity of care index |  |  |  |  |  |
| UPC | 0.955 (0.86-1.07) | 0.42 |  | 0.975 (0.86-1.11) | 0.698 |
| COCI | 1.200 (1.08-1.33) | <0.01 |  | 1.047 (0.92-1.19) | 0.496 |
| SECOC | 0.904 (0.81-1.01) | 0.08 |  | 0.972 (0.85-1.11) | 0.682 |
| PCP type (ref: Attending) |  |  |  |  |  |
| Resident | 0.859 (0.76-0.98) | 0.02 |  | 0.845 (0.72-0.99) | 0.03 |
| Advanced practitioner | 1.047 (0.96-1.14) | 0.28 |  | 0.978 (0.89-1.07) | 0.64 |
| Year of practice (ref: ≤5) |  |  |  |  |  |
| 6-10 | 0.960 (0.87-1.06) | 0.41 |  | 0.987 (0.88-1.10) | 0.81 |
| 11-15 | 0.839 (0.75-0.93) | <0.01 |  | 0.916 (0.81-1.03) | 0.16 |
| 16+ | 0.822 (0.74-0.91) | <0.01 |  | 0.852 (0.76-0.95) | <0.01 |
| Distance: Home to clinic | 1.004 (1.001-1.01) | <0.01 |  | 1.004 (1.002-1.01) | <0.01 |
| Rural | 0.904 (0.83-0.98) | 0.02 |  | 0.921 (0.84-1.00) | 0.06 |
| ADI: Nation Ranking | 1.004 (1.002-1.01) | <0.01 |  | 1.001 (0.99-1.01) | 0.29 |

Prior visits: Numbers of completed visits, no-shows, and same day cancellation

Comorbidities: Numbers of comorbidities

UPC: Usual provider of care index; COCI: Continuity of care index; SECOC: Sequential continuity of care index

Advanced practitioner: Nurse practitioners and physician assistants

Distance: Distance in miles from a patient’s home to the patient’s clinic

ADI: Area deprivation index
